# Genome-wide characterisation and expression analysis of *PLATZ* gene family of *Eucalyptus grandis* under arbuscular mycorrhizal symbiosis

**DOI:** 10.3389/fpls.2026.1727363

**Published:** 2026-03-23

**Authors:** Qiutong Liu, Xinyi Fan, Jianlang Zhang, Xinzhu Yang, Jinquan Liu, Kexin Li, Zujing Chen

**Affiliations:** 1 College of Forestry and Landscape Architecture, South China Agricultural University, Guangzhou, China; 2 State Key Laboratory of Conservation and Utilization of Subtropical Agro-Bioresources, Guangdong Laboratory for Lingnan Modern Agriculture, South China Agricultural University, Guangzhou, China

**Keywords:** *Arbuscular mycorrhizal*, *Eucalyptus grandis*, gene expression, genome-wide identification, PLATZ gene family

## Abstract

**Introduction:**

Arbuscular mycorrhizal fungi (AMF) affect plant performance and ecosystem functioning. The Plant AT-rich protein and zinc-binding protein (*PLATZ*) transcription factor family plays a pivotal role in the plant growth and meristem activity. However, the potential role of *PLATZ* genes in regulating arbuscular mycorrhizal (AM) symbiosis remains experimentally uncharacterized.

**Methods:**

This study aimed to identify the *PLATZ* genes in *Eucalyptus grandis* and to preliminarily characterize their dual involvement in both growth regulation and AM symbiosis. A genome-wide identification and expression analysis of *EgPLATZ* genes was conducted through bioinformatics approaches and transcriptomic data.

**Results:**

Twenty *EgPLATZ* members were identified and classified into 4 distinct clades, which is consistent with conserved domain architectures observed in other plant species. *EgPLATZs* are enriched in cis-regulatory motifs associated with cell expansion, phytohormone signaling, meristem activity, and mycorrhizal symbiosis, especially NODCON2GM (100%), PIBS (50%) and AW-box (75%). The protein predictions suggest that *EgPLATZ* proteins interact with Dof proteins and transcription initiation factors, indicating a conserved transcriptional mechanism analogous to that observed in other species. *EgPLATZs* exhibit tissue-specific expression patterns, and *EgPLATZ14* and *EgPLATZ15* were highly expressed in roots and leaves, respectively. Six *EgPLATZs* were generally down-regulated under AM symbiosis, with *EgPLATZ15* and *EgPLATZ2* showing significant downregulation.

**Discussion:**

These results suggest that certain *EgPLATZs* may function at the interface of developmental signaling and mycorrhizal colonization. This study provides the genome-wide characterization of *PLATZs* in *E. grandis*, establishing a functional framework for future investigations into their roles in growth and symbiosis, and suggestion potential candidate genes involved in AMF-responsive in *E. grandis*.

## 1 Introduction

*Eucalyptus grandis*, belonging to the Myrtaceae family of dicotyledonous plants, are mainly distributed in subtropical and tropical areas (Martin and Van Der Heijden, 2024). This species are widely cultivated worldwide with remarkable adaptation, wide uses, fast growth, great economic and environmental value and superior wood properties (Myburg et al., 2014; Zhang and Wang, 2021; Liu et al., 2023a). However, the rapid biomass accumulation of *E. grandis* is frequently impeded by low bioavailable soil phosphorus and suboptimal root system architecture (De Oliveira et al., 2022; Fang et al., 2024; Li et al., 2025). The restricted development of lateral roots and root hairs frequently compromises the plant’s capacity for efficient soil nutrient exploration (Ferreira et al., 2021; Kuang et al., 2023). Therefore, enhancing the root development and promoting nutrient absorption is crucial for *E. grandi* to enhance the survival fitness and productivity.

*E. grandis* has evolved a mutualistic symbiosis with arbuscular mycorrhizal fungi (AMF). AMF have formed a coevolutionary relationship with approximately 80% of terrestrial plants for up to 450 million years (Bonfante and Venice, 2020; Genre et al., 2020). AMF establishes close physiological connections with host plants through arbuscules within the roots, forming a “plant-fungal reciprocal network” (Lethielleux-Juge, 2025; Zeng et al., 2025). This significantly enhance nutrient acquisition efficiency and abiotic stress tolerance (Devi et al., 2023; Mbodj et al., 2025). However, recent studies have revealed the complex molecular dialogue mechanism between AMF and plant hosts (Martin and Van Der Heijden, 2024; Lethielleux-Juge, 2025). This process is precisely orchestrated by a highly coordinated transcriptional regulatory network that governs both host cellular reprogramming and symbiotic signal transduction (Ho-Plágaro and García-Garrido, 2022; Shi et al., 2023). For instance, plants attract AMF mycelium by secreting signaling molecules such as strigolactones, and AMF activates the symbiotic signaling pathways of plants through lipid chitin oligosaccharides, ultimately facilitating the formation of symbiotic structures (Ito et al., 2022). AMF maintain hormonal balance by inducing a substantial increase in IAA and cytokinin levels while reducing ABA levels in both roots and leaves after root damage. This hormonal regulation results in an increased proportion of fine roots and effectively promotes overall root growth (Zhang et al., 2021; Begum et al., 2022). Transcription factors (TFs) such as GRAS, MYB, and AP2/ERF have been identified as central regulators of this symbiotic process (Xie et al., 2022; Shi et al., 2023). Therefore, it is critical to understand the identities and functional roles of transcription factors that orchestrate these symbiotic processes.

TFs constitute represent a fundamental class of transcriptional regulators that orchestrate diverse physiological processes, and a comprehensive investigation of TFs will facilitate a deeper understanding of their evolutionary origins, biological functions, and regulatory mechanisms (Sun et al., 2021; Liu et al., 2022; Strader et al., 2022; Zuo et al., 2023). PLATZ (Plant AT-rich sequence and zinc-binding) is a type of transcription factor specific to plants. The first member of PLATZ, PLATZ1, was isolated from peas, and these proteins contained relatively conserved cysteine and histidine residues (Nagano, 2001). The PLATZ transcription factors harbor two separated zinc finger domains, namely C-x_2_-H-x_11_-C-x_2_-C-x_(4-5)_-C-x_2_-C-x_(3-7)_-H-x_2_-H and C-x_2_-C-x_(10-11)_-C-x_3_-C, which were necessary for zinc-dependent DNA binding (Zhang et al., 2023). It can non-specifically bind to sequences rich in A/T and plays a role in transcriptional inhibition (Nagano, 2001). The *PLATZ* gene family has been identified in several plant species (Li et al., 2023; Wang et al., 2024).

The PLATZ protein and *PLATZ* genes can regulate the growth and responses to various abiotic stresses in various plants (Wai et al., 2022; Baucher et al., 2024). In *A. thaliana* and *P. trichocarpa*, a molecular link connecting leaf development with either ORE15 or *PtrPLATZ14* through the growth regulating factor-GRF interacting factor (GRF-GIF) regulatory system (Jun et al., 2019; Wang et al., 2024b). Overexpression of *AtPLATZ2* in wild-type *A. thaliana* significantly enhances their sensitivity to salt stress (Liu et al., 2020). Furthermore, the *PLATZ* family has emerged as a critical regulator of root apical meristem (RAM) activity and cellular proliferation. Studies have identified regulatory relationships between root development and specific members of the *PLATZ* gene family in *A. thaliana*, including ORE15 and RITF1 (Zhang et al., 2025). ORE15 integrates auxin signaling to enhance the spatial branching capacity of the root system (Kim, 2019; Timilsina et al., 2022). RITF1 act as a pivotal downstream effector of peptide hormone signaling to ensure sufficient cell proliferative potential within the RAM (Yamada et al., 2020). They are essential for maintaining the balance between cell division and differentiation in roots, revealing their conserved roles as transcriptional regulators in this developmental process (Yamada et al., 2020; Timilsina et al., 2022; Zhang et al., 2025). Such transcriptional regulation of root cellular proliferation and differentiation likely provide a developmental framework that facilitates arbuscule formation by AMF (Xie et al., 2022). Therefore, characterizing the PLATZ transcription factor family in *E. grandis* under AMF inoculation is essential to bridge the mechanistic gap between transcriptional regulation and symbiotic optimizatio.

Based on the evolutionarily conserved roles of *PLATZ* genes in root development and stress adaptation, we hypothesize that specific *EgPLATZ* genes members may modulate the *Eucalyptus*-AMF symbiosis by regulating host root architectural traits or symbiotic signal transduction. With advances in the availability of the whole *E. grandis* genome sequences, it has become feasible to identify the *PLATZ* gene family in *E. grandis* on a genome-wide scale. This study describes the *PLATZs* from the following aspects: (1) physicochemical property, chromosome distribution and gene replication analysis of the *EgPLATZ* gene based on the latest *E. grandis* genomic data; (2) phylogenetic development, multiple sequence alignment and collinearity analysis of *EgPLATZ* family; (3) analysis of the cis-acting elements and protein-protein interaction network of *EgPLATZs*; (4) the RNA-seq data from AMF-mycorrhizal and non-mycorrhized *E. grandis* and the expression locations of candidate *EgPLATZ* genes.

## 2 Materials and methods

### 2.1 Identification and phylogenetic of PLATZ in the *E. grandis* genome

Based on the *E. grandis* genome sequences and annotation files received from the NCBI nucleotide sequence repository (https://www.ncbi.nlm.nih.gov/datasets/taxonomy/71139/), he reference genome used in this study was ASM1654582v1, released in January 2021, and the hidden markov model (HMM) profiles corresponding to the PLATZ domain (PF04640) downloaded from the PFAM database (https://pfam.xfam.org/) (Finn et al., 2014), we use a basic HMM search by TBtools v2.086 to identify PLATZ family proteins in the *E. grandis* protein database with an e-value cutoff of 1 × 10^-5^ (Chen et al., 2023). For genes with multiple alternative splicing isoforms, only the longest protein sequence was retained to avoid redundancy in downstream analyses (Zhang et al., 2020). Although the results all have complete or partial PLATZ domain, the sequences may contain putative *EgPLATZ* which are pseudogenes, incomplete assemblies, sequencing errors, or mispredictions. To eliminate the error caused by wrong prediction, 13 *Arabidopsis* PLATZ protein sequences were searched by BLASTP in *E. grandis* protein database and candidate sequences were obtained (Schaffer, 2001). Submitting the results of the HMM retrieval and BLASTP operations to the NCBI CDD (http://www.ncbi.nlm.nih.gov/cdd/) and SMART databases (http://smart.embl.de/) confirmed the existence of PLATZ domain in all *Eucalyptus* PLATZ protein sequences. Members of the PLATZ gene family were initially identified based on the presence of the conserved PLATZ domain. The conserved domains of these proteins were validated using SMART and NCBI CDD databases. After manual inspection, proteins with incomplete domains were excluded, resulting in a total of 20 PLATZ members. The ExPASy website (https://web.expasy.org/compute_pi/) was used to analyze the *EgPLATZ* gene sequences to obtain the theoretical isoelectric points (pIs) and molecular weights (MWs) (Gasteiger, 2003). And the PSORT online web site (https://wolfpsort.hgc.jp/) was used for the *EgPLATZ* family members subcellular localization prediction.

### 2.2 Chromosomal distribution, gene structure and conserved domain

Utilizing the TBtools software based on the genome annotation information of *E. grandis*, the *EgPLATZ* genes were mapped onto the chromosomes, and the chromosomal positions of the *PLATZ* genes of *E. grandis* were analyzed (Wai et al., 2022). Structural domains were identified by submitting gene sequences to the Batch CD-Search tool on the NCBI website. Finally, the Gene Structure View function in TBtools was used to visualize and integrate the structural domains as well as the exon-intron organization of each gene (Guan et al., 2024).

### 2.3 Phylogenetic tree analysis and multiple sequence alignment

In order to investigate the phylogenetic relationship of the PLATZ transcription factors in *E. grandis*, *A. thaliana*, *P. trichocarpa*, and *O. sativa*, the amino acid sequences of the members of the *EgPLATZ*, *AtPLATZ*, *PtPLATZ*, and *OsPLATZ* gene families were employed for the construction of the phylogenetic tree, the amino acid sequence data for PLATZ proteins in *A. thaliana*, *P. trichocarpa*, and *O. sativa* were obtained from the Phytozome database (https://phytozome-next.jgi.doe.gov/). The MUSCLE module in the MAGE software was utilized for sequence alignment (Tamura et al., 2021). According to the sequence alignment results, the phylogenetic tree was constructed using the neighbor-joining (NJ) method. A bootstrap evaluation was conducted on the phylogenetic tree, with the iteration number (Bootstrap) set at 1000, and other parameters were set to default values. The resulting phylogenetic tree was visualized and annotated using the Interactive Tree of Life (iTOL) platform (https://itol.embl.de/). Multiple sequence alignment of the EgPLATZ protein was conducted using DNAMAN software (Xu et al., 2025).

### 2.4 Collinearity analysis, cis-element analysis and protein-protein interaction network

Chromosome length data for *E. grandis* were first compiled using the Fasta Stats module in TBtools v2.086 to generate the reference file (ChrLen.txt) (Chen et al., 2023). Intra-species gene relationships were then identified using the One-Step MCScanX–Super Fast module, and syntenic relationships within the *E. grandis* genome were visualized with the Advanced Circos module. Finally, the Dual Synteny Plotter program in TBtools was used to analyze and illustrate chromosomal collinearity in *E. grandis.* For comparative evolutionary analysis, inter-species collinearity between *E. grandis* and the reference species *A. thaliana* and *O. sativa* was assessed using the same MCScanX module. Chromosome nomenclature was standardized across species in the output CTL files, and the results were visualized using the Dual Synteny Plot for MCScanX module in TBtools (Nie et al., 2025). The upstream 2 kb sequence was extracted as the promoter region for the prediction of cis-acting elements using the online tool PlantCARE (http://bioinformatics.psb.ugent.be/webtools/plantcare/html/), and the predicted results were Statistics collated by Excel and drawn by Origin 2021 (Lescot, 2002). Then, Simple BioSequence Viewer program in TBtools was utilized for generating the circos visualization. The protein-protein interaction network diagram (PPI) was generated by performing a search based on the “protein by sequences” option in the search function on the STRING website (https://cn.string-db.org), with the minimum interaction score set at 0.400. Analysis parameters included full STRING networks, edge evidence annotation, medium confidence for interaction scores (threshold: 0.400), and a maximum of 20 interactors displayed (Xing et al., 2025).

### 2.5 Gene expression analysis

Plant materials consisted of *E. grandis* seeds, which were obtained from the Tropical Forestry Research Institute, Chinese Academy of Forestry, Guangdong Province, China. The inoculated AM fungi was *Rhizophagus irregularis* DAOM 197198, from the mycorrhizal biology team, College of Forestry and Landscape Architecture, South China Agricultural University. RNAs from the roots of non-mycorrhizal and mycorrhizal *E. grandis* seedlings were sent to Lianchuan Biotechnology, Hangzhou for cDNA library construction and double-end sequencing of the cDNA library using the Illumina NovaSeq™ 6000 platform (LC-Bio Technologies Co., Ltd., Hangzhou, China). TBtools was used to perform normalization analysis of the FPKM data and to identify significant differences (|log_2_FC ≥ 1| was considered to be significant). The heatmap diagram was also constructed with log_2_ values using TBtools. The expression pattern of *EgPLATZs* was predicted by using website ePlant (https://bar.utoronto.ca) (Waese et al., 2017). We selected the expression pattern diagrams of *EgPLATZ* genes during the 30 days and 6 months of plant growth, and used Photoshop software to summarize.

## 3 Results

### 3.1 Phylogenetic and multiple sequence alignment of the *EgPLATZ* gene family

The physicochemical properties of *EgPLATZ* gene family members were analyzed (**Table 1**). The *EgPLATZ* genes contain 3~4 exons. And the predicted protein products, isoelectric points (pI) and molecular weights (Mw) of the *EgPLATZ* genes varied among members, ranging from 127 (EgPLATZ2) - 298 (EgPLATZ14) aa, 6.99 (EgPLATZ7) - 9.52 (EgPLATZ10), and 14.466 (EgPLATZ2) - 33.723 (EgPLATZ14) kDa, respectively. Among the 20 protein sequences, only EgPLATZ7 possessed a pI below 7.0, suggesting that most EgPLATZ proteins are classified as basic proteins. In silico subcellular localization analysis predicted that all EgPLATZ proteins are localized in the nucleus, while EgPLATZ12 exhibited potential localization to chloroplasts and mitochondria.

**Table 1:** The PLATZ family genes in *E. grandis*.

Gene name	Gene ID	Exons	Protein			Predicted localization
			Length (aa)	pI	MW (kDa)	
*EgPLATZ1*	rna-XM_010051988.3	3	210	9.50	23.838	Nucleus
*EgPLATZ2*	rna-XM_039306451.1	3	127	7.61	14.466	Nucleus
*EgPLATZ3*	rna-XM_010048189.2	3	215	9.20	24.436	Nucleus
*EgPLATZ4*	rna-XM_010046984.3	3	224	8.89	25.413	Nucleus
*EgPLATZ5*	rna-XM_010048899.3	3	260	8.70	29.453	Nucleus
*EgPLATZ6*	rna-XM_039309628.1	3	159	8.99	18.298	Nucleus
*EgPLATZ7*	rna-XM_010053542.3	4	264	6.99	29.921	Nucleus
*EgPLATZ8*	rna-XM_039311538.1	3	198	9.19	22.342	Nucleus
*EgPLATZ9*	rna-XM_039311539.1	3	187	8.94	21.291	Nucleus
*EgPLATZ10*	rna-XM_010055315.3	3	254	9.52	28.552	Nucleus
*EgPLATZ11*	rna-XM_010057014.3	3	233	9.49	26.535	Nucleus
*EgPLATZ12*	rna-XM_010057221.3	4	249	9.48	27.809	Chloroplast. Mitochondrion. Nucleus.
*EgPLATZ13*	rna-XM_010058061.3	4	247	8.92	27.607	Nucleus
*EgPLATZ14*	rna-XM_010059751.3	4	298	8.73	33.723	Nucleus
*EgPLATZ15*	rna-XM_010063038.3	3	243	9.12	27.397	Nucleus
*EgPLATZ16*	rna-XM_010064883.3	3	248	8.89	27.881	Nucleus
*EgPLATZ17*	rna-XM_010068252.3	3	228	9.20	25.626	Nucleus
*EgPLATZ18*	rna-XM_039302454.1	3	203	9.09	22.954	Nucleus
*EgPLATZ19*	rna-XM_010036156.2	3	198	8.97	22.611	Nucleus
*EgPLATZ20*	rna-XM_039302884.1	3	164	9.07	18.536	Nucleus

Column 1 lists gene nomenclature; Column 2 provides transcript identifiers; Column 3 indicates exon numbers; Columns 4-6 report protein physicochemical properties, including length, isoelectric point (pI), and molecular weight; and Column 7 specifies the predicted subcellular localization.

### 3.2 Chromosomal mapping and structural analysis of *EgPLATZ* genes

**Figure 1:**
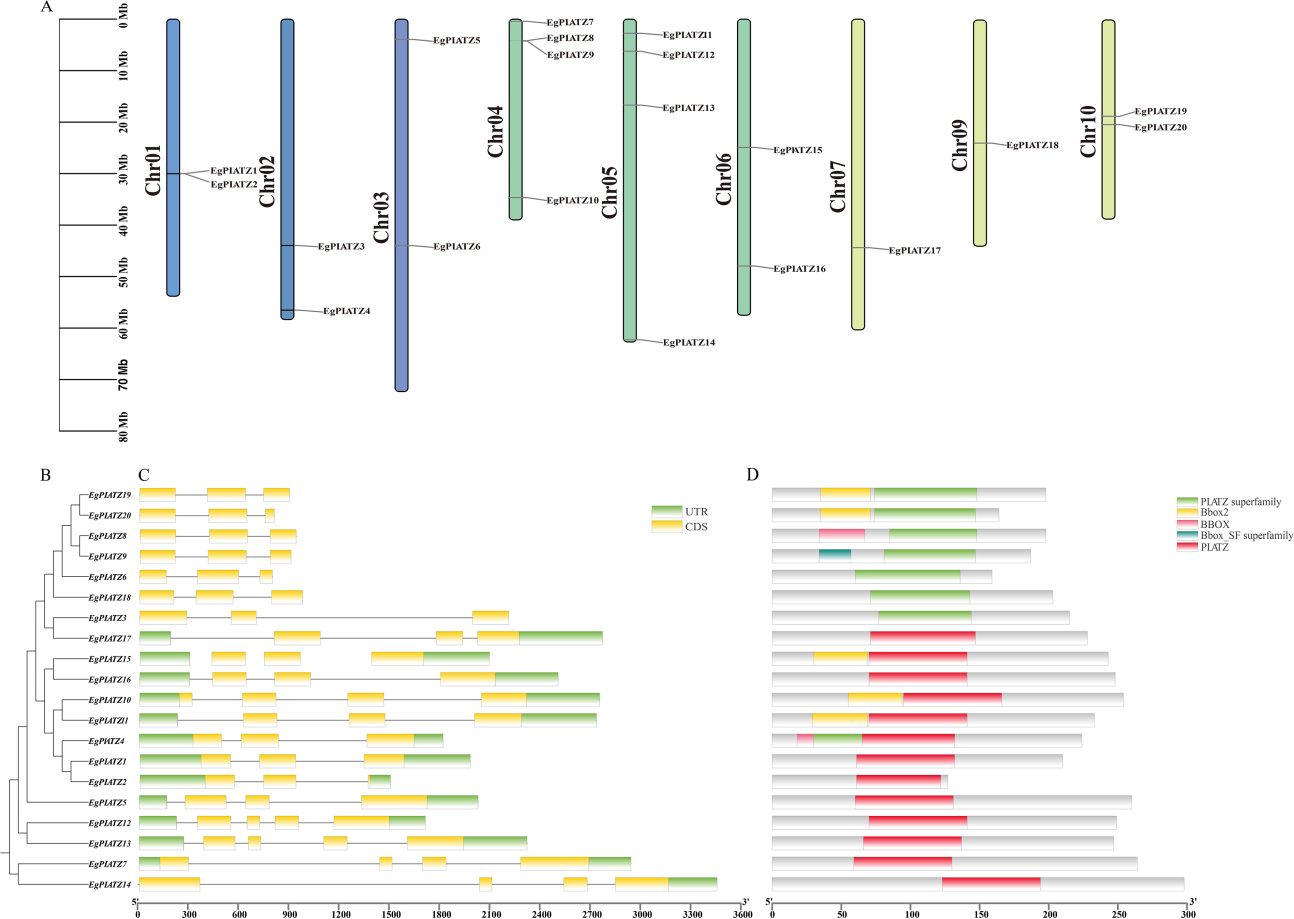
Schematic diagram of the chromosomal distribution, conserved domain and gene structure of *EgPLATZ* genes in *E. grandis*. **(A)** chromosomal distribution. the vertical bars represent the chromosomes of *Eucalyptus*. **(B)** Phylogenetic tree within *E. grandis*. **(C)** The exon-intron structures of the *EgPLATZs*. UTRs are denoted by green boxes, CDS by yellow boxes, and introns by narrow grey lines. **(D)** Conserved domain of *EgPLATZ*. The legend is shown in the upper-right corner.

**Figure 2:**
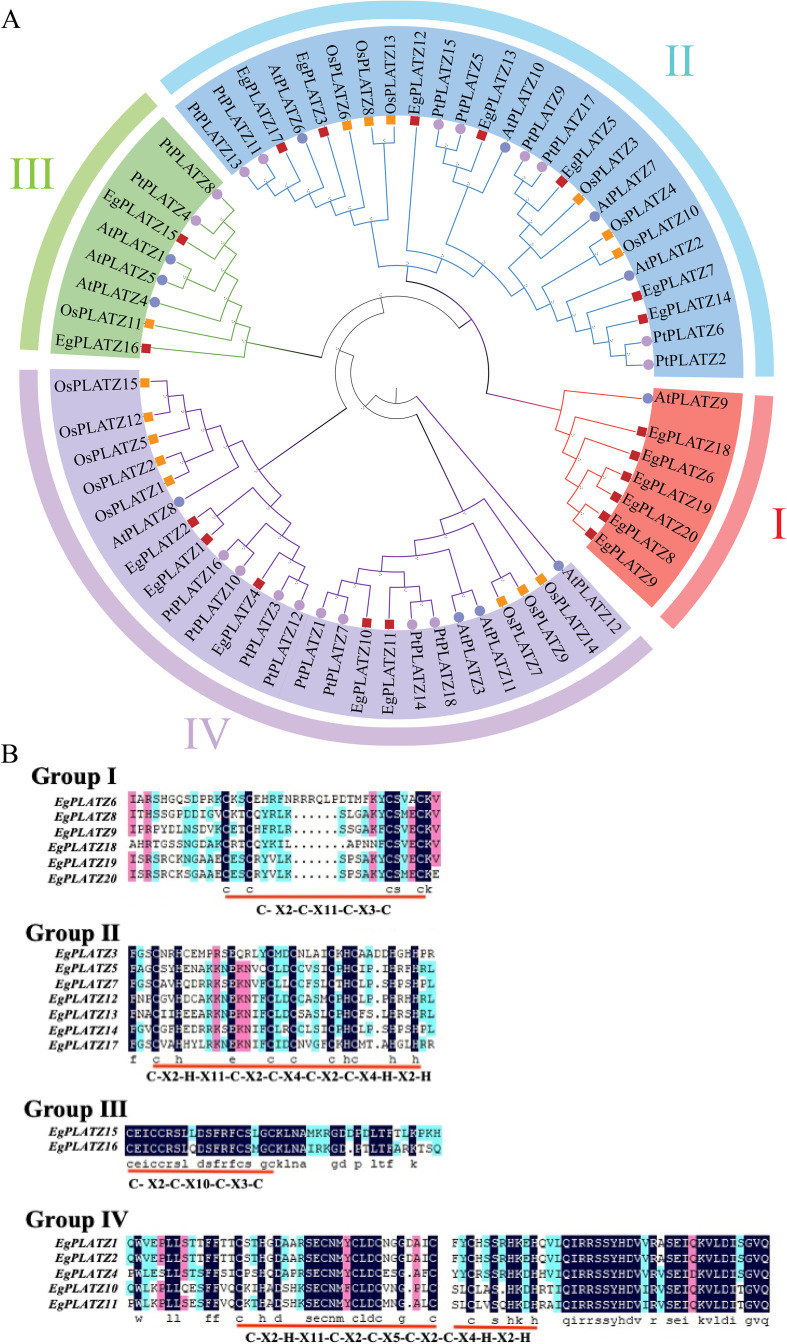
Phylogenetic analysis and multiple sequence alignment of PLATZ proteins. **(A)** Phylogenetic analysis of PLATZ proteins. Colors denoted groups I, groups II, groups III, group IV subfamily. **(B)** Multiple sequence alignment of the conserved domains. The zinc-finger structure is underlined.

A total of 20 candidate sequences were screened in the *E. grandis* genome and designated as EgPLATZ1-EgPLATZ20 based on their chromosomal positions (**Figure 1A**). Except for chromosomes 8 and 11 in *E. grandis*, the 20 *EgPLATZ* genes were unevenly distributed across all chromosomes. The number of *EgPLATZs* was the highest on chromosomes 4 and 5, with four; chromosomes 1, 2, 3, 6 and 10 had two each; chromosomes 7 and 9 had one each. Gene structure analysis (**Figure 1C**) revealed that the *EgPLATZ* genes contain 3~4 exons and 1~3 introns. Consistent with the data of the evolutionary tree (**Figures 1B**, **2A**), the structures of group I (*EgPLATZ3*, *EgPLATZ6*, *EgPLATZ8*, *EgPLATZ9*, *EgPLATZ18*, *EgPLATZ19* and *EgPLATZ20*) are similar. The other 13 genes are similar in structure, but exhibited significant variations in sequence length. Conserved domain analysis (**Figure 1D**) showed that all *EgPLATZ* contains a single PLATZ or PLATZ superfamily conserved domain in the central region, with the notable exception of *EgPLATZ4*, which has two conserved domains simultaneously. This structural configuration differs from that of PLATZ proteins in other plant species such as *Triticum aestivum*, *Medicago sativa*, *O. sativa*, *A. thaliana*, and *P. trichocarpa*, where members typically possess only a single domain independently (**Figure 1D**), suggesting a distinct structural diversification among *EgPLATZ* genes during evolution.

### 3.3 Phylogenetic and multiple sequence alignment of *PLATZ* in the *E. grandis* genome

To elucidate the evolutionary relationship and explore the similarity and diversity of motif compositions, a neighbor-joining phylogenetic tree among *EgPLATZ*, *AtPLATZ*, *PtPLATZ*, and *OsPLATZ* gene families was constructed (**Figure 2A**). The *E. grandis* genome contains 20 *PLATZ* genes, slightly more than *A. thaliana* (13 *AtPLATZs*), *O. sativa* (15 *OsPLATZs*) and *P. tomentosa* (18 *PtPLATZs*). These proteins can be divided into 4 subclasses. Among them, subgroup II contains 7 *EgPLATZ* genes,4 *AtPLATZ* genes, 6 *OsPLATZ* genes and 8 *PtPLATZ* genes; The IV subgroup contains 5 *EgPLATZ* genes, 4 *AtPLATZ* genes, 8 *OsPLATZ* genes and 8 *PtPLATZ* genes. The *EgPLATZs* are mostly present in group I, II and IV. Notably, 12 *EgPLATZs* exhibited higher sequence homology with PLATZ members from Arabidopsis, rice, and poplar within groups II and IV. And there is only *AtPLATZ9* and six *EgPLATZs* under Group I, suggestinging that the evolution of *EgPLATZ6*, *EgPLATZ8*, *EgPLATZ9*, *EgPLATZ18*, *EgPLATZ19* and *EgPLATZ20* are relatively conserved (**Figure 2A**).

Combining the phylogenetic tree with the zinc finger structure analysis, the *EgPLATZs* were further classified into four subgroups (**Figure 2B**). Sequence motif analysis revealed that the group II and IV members harbor five cysteine and three histidine residues [C-x2-H-x11C-x2-C-x(4-5)-C-x2-C-x(3-5)-H-x2-H], whereas the group I and group III regions are with four cysteine residues [C- x2-C-x(10-12)-C-x3-C].

### 3.4 Collinearity analysis of *EgPLATZ* genes

**Figure 3:**
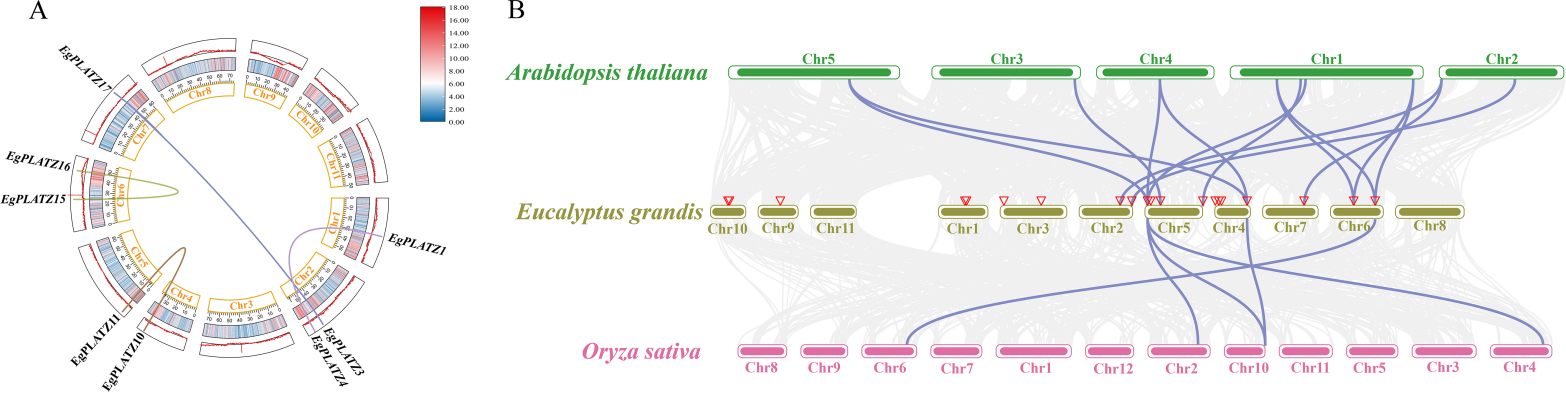
Circos plots of the chromosomal locations of *EgPLATZs* with duplication links. **(A)** Duplication of *EgPLATZs* within the *E*. *grandis* genome. **(B)** Duplication of *EgPLATZ* orthologs within the *A. thaliana* and *O. sativa* genome. A pair of genes with the same color connected by a line represents a duplication relationship.

To investigate the expansion of the *EgPLATZ* gene family, collinear analysis on 20 *EgPLATZ* genes was performed (**Figure 3**). Four segmental duplication events were identified within the *EgPLATZ* gene family, while no tandem duplication events were detected (**Figure 3A**). This suggests that segmental duplication events are the primary drivers of family expansion in *E. grandis*. Four pairs of segmentally duplicated genes are *EgPLATZ1* (Chr1) & *EgPLATZ4* (Chr2), *EgPLATZ3* (Chr2) & *EgPLATZ17* (Chr7), *EgPLATZ10* (Chr4) & *EgPLATZ11* (Chr5), and *EgPLATZ15* (Chr6) & *EgPLATZ16* (Chr6). These gene pairs exhibit a one - to - one correspondence. Additionally, there are 14 pairs of *PLATZ* homologous genes between *E. grandis* and *A. thaliana*, which are located on chromosomes2, 4, 5, 6, 7, and 5 pairs of *PLATZ* homologous genes between *E. grandis* and *O. sativa*, which are located on chromosomes4, 5, 6 (**Figure 3B**). Some homologous gene pairs only occur in dicotyledonous plants (*A. thaliana*), such as *EgPLATZ3*, *EgPLATZ4*, *EgPLATZ14*, *EgPLATZ15* and *EgPLATZ17*.

### 3.5 Cis-acting element analysis and protein-protein interaction network of EgPLATZs

**Figure 4:**
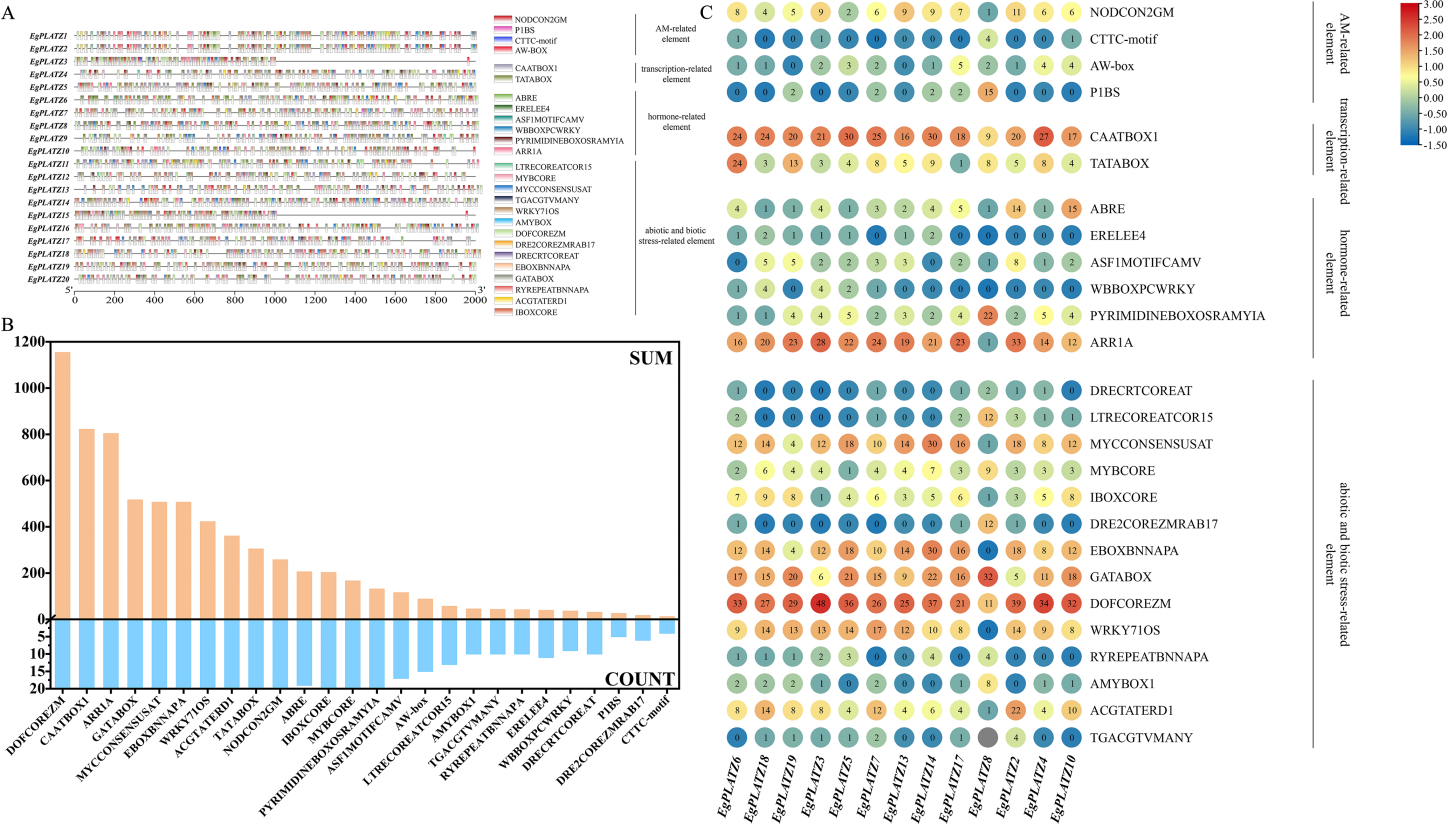
Identification of cis-acting elements in the promoter regions of *EgPLATZs*. **(A)** The distribution of cis-acting elements in different *EgPLATZs*. Different colors correspond to different members of the group. **(B)** the total quality of Cis-acting elements. SUM represents the total number of cis-acting elements upstream of the 20 *EgPLATZs*. COUNT represents the number of *EgPLATZs* whose upstream regions contain the respective elements. **(C)** Number of cis-acting elements in *EgPLATZs* that are typical distribution in different subfamilies.

A total of 26 cis-acting elements were identified in the 2,000 bp upstream regions of *EgPLATZs*. These elements were categorized into four functional groups: AM symbiotic relationships, transcription regulation, phytohormone response and stress response (**Figures 4B, C**). Regarding transcription, the CAATBOX1 and TATABOX cis-acting elements were distributed in all *EgPLATZs*, with CAATBOX1 being the most abundant (**Figures 4B, C**). For abiotic and biotic stress responses, elements related to light, drought, low temperature, wound-responsive, and general stress were present. These are similar to the characteristics of cis-acting elements of PLATZ TFs in other species. Hormone-responsive elements were also prevalent, with over 80% of *EgPLATZ* members containing the cis-acting elements related to abscisic acid (ABRE), gibberellic acid (PYRIMIDINEBOXOSRAMYIA), cytokinin response (ARR1A).

Additionally, Several cis-acting elements associated with mycorrhizal symbiosis were identified in *EgPLATZs*, such as NODCON2GM, P1BS, CTTC-motif, and AW-box (Shi et al., 2023; Duan et al., 2024). Among these, NODCON2GM exhibited the highest frequency (257 occurrences) and distributed in all *EgPLATZ* genes. AW-box, P1BS, and CTTC-motif were found in 75%, 25%, and 20% of the members, respectively (**Figure 4B**). And these four symbiosis-related cis-acting elements are predominantly distributed in group II and IV (**Figure 4A**).

**Figure 5:**
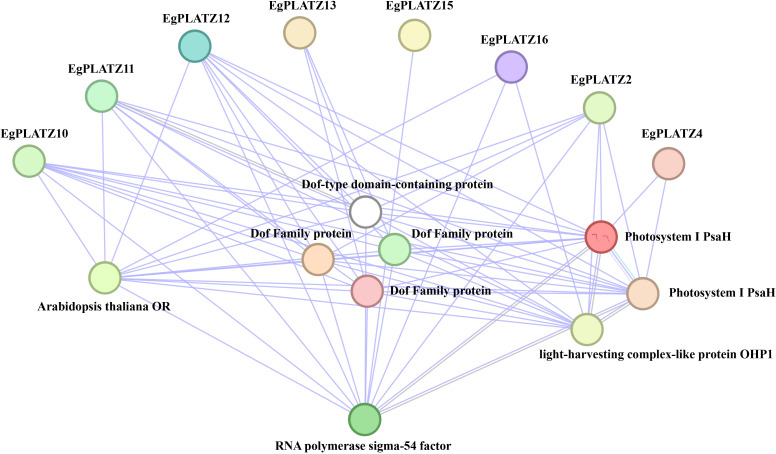
The interaction network of EgPLATZ family proteins and target proteins. Nodes represent different proteins, and lines represent the interactions between proteins.

To further explore the potential regulatory network, a protein-protein interaction (PPI) network was constructed (**Figure 5**).The prediction suggested that EgPLATZ family proteins exhibit potential interactions with a diverse range of proteins. Specifically, multiple members, including EgPLATZ2, EgPLATZ 4, EgPLATZ10, EgPLATZ 11, EgPLATZ12, EgPLATZ13, EgPLATZ15, and EgPLATZ16, were predicted to interact with Dof-type domain-containing proteins and several other Dof family members. And potential interactions were observed between PLATZ proteins and components associated with the photosynthetic system and RNA polymerase, such as Photosystem I PsaH, the light-harvesting complex-like protein OHP1 and sigma-54 factor. Simultaneously, EgPLATZ2, EgPLATZ10, EgPLATZ11, EgPLATZ12, and EgPLATZ16 exhibited predicted associations with *A. thaliana* orange (OR) proteins (**Figure 5**).

### 3.6 Gene expression analysis of *EgPLATZs*

**Figure 6:**
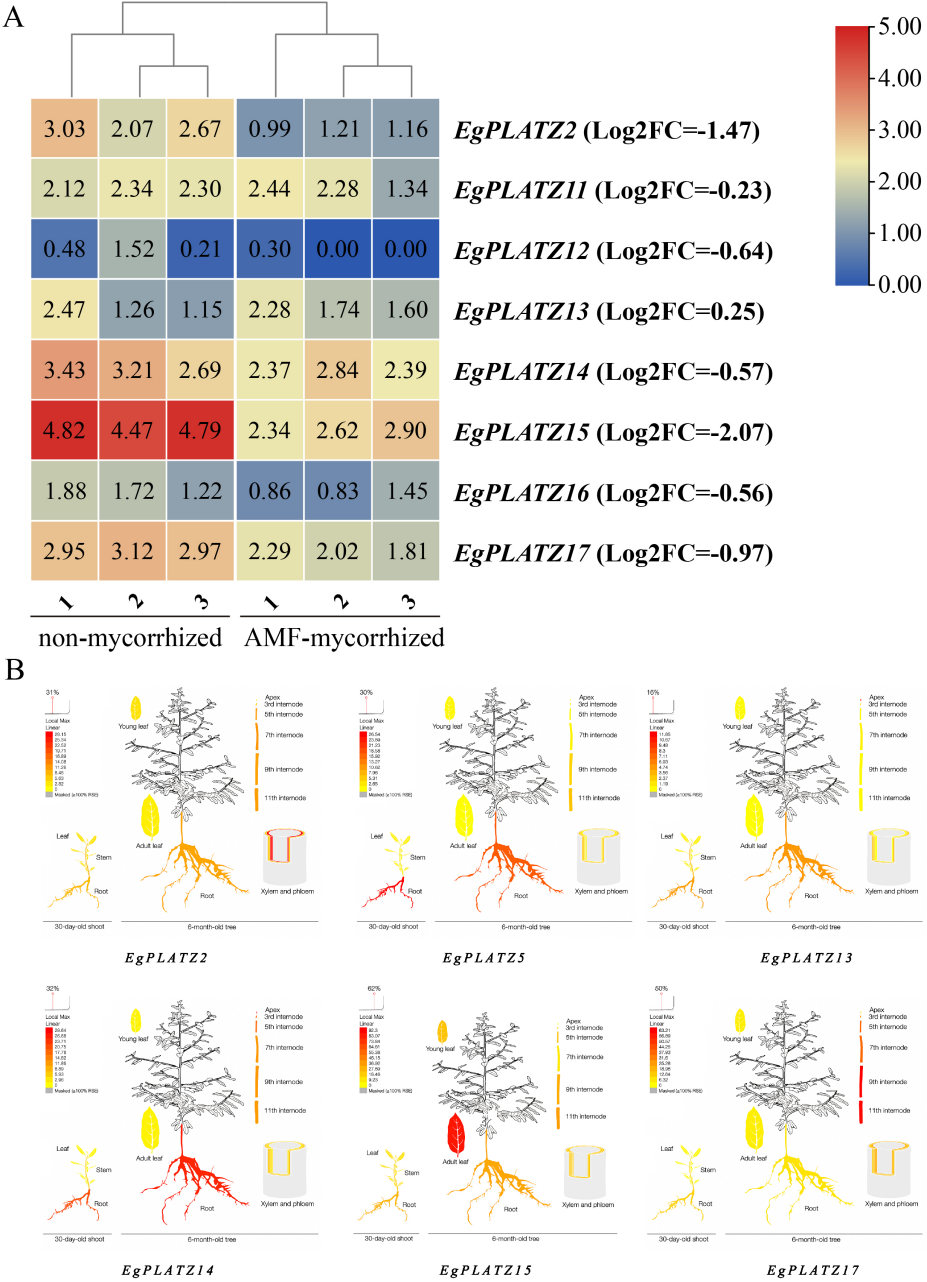
Heatmap of *EgPLATZ* gene expression determined by RNA-seq. Log_2_-fold differences in gene expression (FPKM values) were used to create the heatmap. **(A)** Heatmap showing the expression levels of EgPLATZ genes in non-mycorrhized and AMF-mycorrhized conditions. **(B)** Expression pattern diagrams of six *EgPLATZ* genes (*EgPLATZ2, EgPLATZ5, EgPLATZ13, EgPLATZ14, EgPLATZ15*, and *EgPLATZ17*) at 30 days and 6 months of plant growth.

The *EgPLATZ* genes were differentially expressed between AMF-mycorrhizal (AM) and non-mycorrhized (NM) roots of *E. grandis* (**Figure 6A**). The expression level of *EgPLATZ15* is the highest in the NM treatment,. Compare to the NM treatment, five genes were downregulated with AM treatment, including *EgPLATZ15* (Log_2_FC=-2.07), *EgPLATZ2* (Log_2_FC=-1.47), *EgPLATZ17* (Log_2_FC=-0.97), *EgPLATZ12* (Log_2_FC=-0.64), *EgPLATZ14* (Log_2_FC=-0.57), *EgPLATZ16* (Log_2_FC=-0.56).

Combined with the results of the phylogenetic tree groups, the heat map of gene expression and the number of cis-acting elements, six candidate genes were selected for further spatio-temporal analysis. The results showed that all *EgPLATZ* genes were expressed in the root of at 30 days of *E. grandis* (**Figure 6B**). At 6 months, *EgPLATZ10*, *EgPLATZ11* and *EgPLATZ15* exhibited higher transcript levels in leaves than in roots. As *E. grandis* grows, over 50% of *EgPLATZ* genes may be upregulated in the root system. And *EgPLATZ15* may exhibit the highest expression level and growth rate.

## 4 Discussion

The PLATZ, a plant - specific transcription factor, plays a crucial role in plant growth, meristem activity, and responses to stresses (Han et al., 2022; Zhang et al., 2025b). While this family has been extensively characterized in several model plants like *A. thaliana* and *O. sativa.*, a systematic identification within *E. grandis* has has remained elusive until now. Moreover, the potential involvement of *PLATZ* gene family during the interaction between *E. grandis* and AMF remains largely uncharacterized. In this study, based on the genomic data of *E. grandis*, a comprehensive and systematic identification of *PLATZ* gene family in *E. grandis* was performed. Specifically, we placed a particular emphasis on analyzing the expression patterns of *EgPLATZ* genes during symbiotic interactions. These findings offer novel insights into the functions of *EgPLATZs* within the context of symbiotic interactions, which is importance for understanding the molecular mechanisms underlying the plant - AMF symbiosis.

The *EgPLATZ* gene family in *E. grandis* comprises a larger number of members than those in model plant species, including *A. thaliana*, *O. sativa*, and *P. trichocarpa*, a variation likely driven by the complex evolutionary history of the eucalyptus genome (Myburg et al., 2014; Lai et al., 2020). A notable finding is the unique structural architecture of *EgPLATZ4*, which contains two tandemly arranged PLATZ domains. This rare configuration not typically observed in orthologs from *T. aestivum*, *M. sativa*, *O. sativa*, *A. thaliana*, and *P. trichocarpa* (Fu et al, 2020; Wai et al., 2022; Li et al., 2023; Wang et al., 2024b). The emergence of multi-domain architectures within this transcription factor family is suggestive of enhanced functional versatility (Lallemand et al, 2020; Wu et al, 2026). This structural innovation in *E. grandis* may reflect an evolutionary adaptation to its distinctive dual-mycorrhizal ecology. Unlike many model plant species, *E. grandis* is a woody perennial capable of establishing symbiotic associations with both AM and ectomycorrhizal (ECM) fungi. Consistent with this, collinearity analysis confirmed that segmental duplication was the predominant driver for the expansion of the *EgPLATZ* family. The occurrence of fragment duplication is often considered to be related to the polyploidization of plants (Jiao et al., 2011). The ratio of the number of PLATZ homologous gene pairs between *E. grandis* and *A. thaliana*/*O. sativa* is 14: 5, indicating that there are certain differences in the evolution of the *PLATZ* genes between monocotyledonous plants and dicotyledonous plants. The homology of the *EgPLATZs* is higher in dicotyledonous plants. The higher degree of homology with dicotyledonous species provides a comparative framework for hypothesizing the potential biological roles of *EgPLATZs.* Specifically, based on their close phylogenetic relationships with well-characterized *AtPLATZ* members, several *EgPLATZ* candidates are putative regulators of stress response and development. The *AtPLATZ1*, which is homologous to *EgPLATZ15* and *EgPLATZ16*, has been shown to enhance drought tolerance by regulating the ABA signaling pathway; the *AtPLATZ3* (ORE15), which is homologous to *EgPLATZ11*, is known to work in concert with GRF (growth-regulation factor) and GIF (grf-interacting factor) to promote cell division and delay leaf senescence; the *AtPLATZ11*, which is homologous to *EgPLATZ10* and *EgPLATZ11*, plays a significant role in the plant’s defense against certain biotic or abiotic stresses; and the *AtPLATZ10*, which is homologous to *EgPLATZ13*, plays a crucial role in the negative regulation of plant tissue and organ development and cell metabolism (Kim et al., 2018; Liu et al., 2020; Fan et al., 2025). While these homologous relationships suggest that *EgPLATZs* may possess conserved functions in *E. grandis*, these inferences remain speculative and necessitate further direct functional validation to confirm their specific roles in eucalyptus. These gene duplication events and the distinctive structural variations likely contributed to the functional diversification of *EgPLATZs*.

PPI analysis suggests that EgPLATZ family members potentially coordinate with four distinct functional modules: Dof family proteins, photosynthetic system, RNA polymerase and *A. thaliana* OR proteins. Dof transcription factors in *Lotus corniculatus* showed significant upregulation during AM symbiosis (Handa et al., 2015). Dof family proteins, functioning as transcription factors, play crucial roles in plant growth and development, as well as in responses to environmental stresses (Zhou et al., 2020; Liu et al., 2021). This finding implies that EgPLATZs may modulate gene transcription and expression through their interactions with Dof family proteins. Photosystem I, a core component of the photosynthetic electron transport chain, is responsible for electron transfer and the generation of reducing power. And OHP1 is involved in either the structural organization or functional regulation of the photosynthetic system (Li et al., 2019; Tian and Chen, 2024). This suggests that EgPLATZ family proteins might be implicated in plant photosynthesis and have an impact on photosynthetic efficiency. Furthermore, the association with sigma-54 factors, which are critical for transcriptional initiation and the regulation of specialized gene sets, suggests a sophisticated regulatory architecture (Francke et al., 2011; Ma et al., 2021). The *A. thaliana* OR proteins interact with proteins such as TIC20 and TIC40 within the chloroplast TIC core complex (Kato et al., 2020; Yuan et al., 2021). Moreover, as DNAJ-like chaperone proteins, they are also involved in plant carotenoid biosynthesis and the regulation of plastid biogenesis and development processes (Yuan et al., 2021; Oogo et al., 2022). the predicted interaction suggests that *EgPLATZs* might also influence plastid precursor import and inner membrane transport. Collectively, these bioinformatic inferences establish a testable hypothesis for the functional role of EgPLATZ transcription factors in regulating symbiosis-associated gene networks in *E. grandis.*

Cis-acting elements are important molecular switches that control various biological activities such as stress responses and developmental processes in organisms (Soma et al., 2021; Kim et al., 2024). Our analysis revealed that EgPLATZ promoters are significantly enriched in elements sensitive to growth, meristem activity, drought, temperature extremes, and abscisic acid (ABA), consistent with the documented stress-responsiveness of PLATZ genes in other species (Hernández-García et al., 2021; Castro-Camba et al., 2022; Liu et al., 2023b; Sun et al., 2023; Samoluk and Seijo, 2025). It is speculated that the *EgPLATZs* play an important role in the growth and development and stress resistance of *E. grandis.* Additionally, *EgPLATZ* promoters are enriched in mycorrhiza-responsive motifs such as NODCON2GM, P1BS, CTTC, and AW-box. Transcriptome profiling revealed marked down-regulation of *EgPLATZ15*, *EgPLATZ2*, *EgPLATZ17*, *EgPLATZ12*, *EgPLATZ14* and *EgPLATZ16* following AMF. Prior studies have implicated PLATZ in pathogen responses. In kiwifruit, *AdPLATZ1* (Achn020891) expression declines continuously after *Botryosphaeria dothidea* challenge (Wang et al., 2020). In soybean, *GmPLATZ1* is specifically induced by drought, high salinity, and ABA (Zhao et al., 2022). In *A. thaliana*, *AtPLATZ7* restrains root-tip ROS accumulation via the RGF1-RGI1 pathway and is up-regulated under drought and salt stress (Dong et al., 2021; Fan et al., 2025). The involvement of PLATZ genes in symbiosis establishment in woody plants remains unexplored. Meanwhile, existing research has shown that the *AtPLATZ1*, which is homologous to *EgPLATZ15*, is a positive regulator of ABA response (González-Morales et al., 2016; Fan et al., 2025). These findings suggest that *E. grandis* undergoes a complex transcriptional reprogramming of nutrient exchange processes following arbuscular mycorrhizal colonization. Rather than a direct functional requirement, the observed downregulation of specific *EgPLATZ* members(*EgPLATZ15* and *EgPLATZ2*) might reflect a physiological response to the established symbiosis. Combining collinearity, transcriptome data and cis-acting element data, the down-regulation of *EgPLATZs* gene expression after inoculation with AMF, we propose a working hypothesis in which AMF-induced increases in host phosphorus levels potentially trigger the activation of jasmonic acid (JA) or salicylic acid (SA) pathways, subsequently leading to the suppression of abscisic acid (ABA) signaling and the observed downregulation of *EgPLATZ* transcripts. This regulatory feedback loop is hypothesized to assist in balancing symbiotic nutrient exchange and redundant nutrient acquisition pathways, thereby potentially optimizing seedling biomass accumulation. However, it is important to emphasize that this model remains an observational association; further research is required to determine whether the altered expression of *EgPLATZs* is a driver or a consequence of the mycorrhizal relationship.

The expression of *EgPLATZ* varies significantly among tissues, indicating that this family may have multiple functions. In fact, *AtPLATZ1* (Dong et al., 2021) induces the expression of ABA in the roots and controls the growth of the main roots; *AtPLATZ7* and *AtPLATZ2* (Liu et al., 2020)controls the size of root meristematic tissue through ROS signals; *OsPLATZ3* exhibits preferential expression in the tapetal cells during anthers development (Li et al., 2024). Among these *EgPLATZ* genes, expression differences emerged among the genes after 6 months of plant development. *EgPLATZ15*, *EgPLATZ10*, and *EgPLATZ11* were highly expressed in leaves and might be related to leaf development. *EgPLATZ14* and *EgPLATZ5* are highly expressed in the root system and may be related to root development.

This study provides the first systematic characterization of the *PLATZ* gene family in *E. grandis*, providing valuable information for future functional studies. By integrating phylogenetic, structural, and expression analyses, candidate *EgPLATZs* implicated in *E. grandis* development and AM symbiosis was identified. However, because the functional attributions and interaction networks presented herein are derived primarily from bioinformatic predictions and homology-based inference, they should be interpreted with appropriate caution. Due to the current lack of relevant experiments to prove it, the specific regulatory mechanisms of these genes have not yet been clarified. Therefore, the accuracy of the prediction results needs to be verified through molecular biology experiments in the later stage, such as Yeast Two-Hybrid, Electrophoretic Mobility Shift Assays.

## 5 Conclusion

In this study, twenty *EgPLATZ* genes were identified in the genome of *E. grandis* systematically categorized into four evolutionary clades. These proteins, ranging from 127 to 298 amino acids, typically harbor a conserved centrally located PLATZ domain (PLATZ or PLATZ-superfamily). Notably, the identification of a unique dual-domain architecture in EgPLATZ4. Four fragment repetition events in *EgPLATZ* genes, which had a higher genetic homology with the dicotyledonous plant *A. thaliana*. EgPLATZ proteins potentially interact with transcription factors, photosynthetic components, and Dof family proteins, *A. thaliana* OR proteins. These interactions may form a regulatory network that coordinates plant growth, development, and adaptation to environmental cues. *EgPLATZs* expression is developmentally and spatially regulated. At 30 days, all *EgPLATZ* genes may expresse in roots; at six months, *EgPLATZ10*, *EgPLATZ11*, and *EgPLATZ15* accumulate mainly in leaves. Most *EgPLATZs* genes contain cis-acting elements related to cell expansion and adverse stress. Furthermore, *EgPLATZs* contain four cis-acting elements related to mycorrhizae, with 100% of members contained NODCON2GM, 75% contained AW-box, and 50% contained P1BS. Six *EgPLATZ* genes were down-regulated under arbuscular mycorrhizal symbiosis, especially *EgPLATZ15* and *EgPLATZ2*. This study lays the foundation for a deeper understanding of the *EgPLATZ* gene family in terms of its the evolutionary mechanisms, functional characteristics, and potential role in AMF-mycorrhizal treatment.

## Data Availability

The datasets presented in this study can be found in online repositories. The names of the repository/repositories and accession number(s) can be found in the article/supplementary material.
